# Polarized Superradiance from CsPbBr_3_ Quantum Dot Superlattice with Controlled Interdot Electronic Coupling

**DOI:** 10.1021/acs.nanolett.5c00478

**Published:** 2025-04-01

**Authors:** Lanyin Luo, Xueting Tang, Junhee Park, Chih-Wei Wang, Mansoo Park, Mohit Khurana, Ashutosh Singh, Jinwoo Cheon, Alexey Belyanin, Alexei V. Sokolov, Dong Hee Son

**Affiliations:** † Department of Physics and Astronomy, Texas A&M University, College Station, Texas 77843, United States; ‡ Institute for Quantum Science and Engineering, Texas A&M University, College Station, Texas 77843, United States; § Department of Chemistry, Texas A&M University, College Station, Texas 77843, United States; ∥ Center for Nanomedicine, Institute for Basic Science and Graduate Program of Nano Biomedical Engineering, Advanced Science Institute, Yonsei University, Seoul 03722, Republic of Korea

**Keywords:** quantum dots, superradiance, electronic coupling, strong confinement

## Abstract

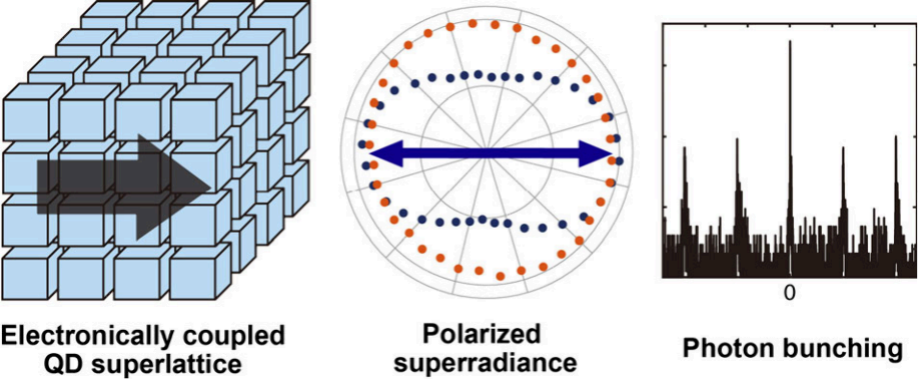

Cooperative emission of photons from an ensemble of quantum dots (QDs) as superradiance can arise from the electronically coupled QDs with a coherent emitting excited state. This contrasts with superfluorescence (Dicke superradiance), where the cooperative photon emission requires a buildup of coherence in an ensemble of incoherently excited QDs via their coupling to a common radiation mode. In perovskite QDs, superradiance has been rarely observed, unlike superfluorescence, due to the challenge in QD electronic coupling. Here, we report superradiance with a very narrow linewidth (<5 meV) and a large redshift (∼200 meV) from the strongly coupled CsPbBr_3_ QD superlattice achieved through the combination of quantum confinement and ligand engineering. The superradiance is polarized in contrast to the uncoupled exciton emission from the same superlattice, indicating anisotropic electronic coupling in superlattices. This finding suggests the potential of a perovskite QD superlattice with structurally controllable interdot coupling as the polarized cooperative photon emitters.

The phenomenon of cooperative emission from an ensemble of dipoles arises from the creation a coherent macroscopic dipole that produces a short burst of spontaneous emission first introduced by Dicke.^1^ In the case of Dicke superradiance, sometimes called superfluorescence, the correlation between optical dipole oscillations of individual emitters is established via their interaction with a common radiation field after the excitation of an incoherent ensemble of absorbers. Therefore, superfluorescence occurs at excitation intensities that can prepare a sufficiently large number of emitters, exhibiting threshold behavior, with a finite delay time required to establish phase coherence among the emitters.^2−5^ However, cooperative emission of photons may also arise from direct electronic coupling between individual emitters, as in the case of J-aggregates in molecular systems.^6−9^ Superradiance from the electronically coupled coherent emitting state can develop without delay, does not exhibit threshold behavior, and should appear at excitation intensities significantly lower than those required for superfluorescence. Furthermore, in superfluorescence (Dicke superradiance), the radiative decay rate can be enhanced by many orders of magnitude up to a factor of *N*, where *N* is the number of quantum emitters that are synchronized.^2,3^ In contrast, in superradiance from electronically coupled emitters, the optical dipole matrix element between the resulting electron bands is determined by the overlap of electron orbitals or the hopping parameter in the tight-binding picture.^10,11^ At the same time, photon bunching is expected as a universal signature of the cooperative emission in any coupling scenario.^2−4,12^ Therefore, electronically coupled systems offer greater flexibility and control over the structure of the emitting states and the properties of the cooperative emission, which are easier to achieve and more robust with respect to decoherence, especially under weak excitations insufficient to produce Dicke superradiance in the absence of electronic coupling.

Metal halide perovskite nanocrystals possess beneficial features as a source of photons such as high luminescence quantum yield and facile tunability of the bandgap.^13,14^ Superfluorescence has been observed from various metal halide perovskite nanocrystals and 2D sheets in recent years.^12,15,16^ More recently, superradiance from the electronically coupled ensemble of perovskite nanocrystals has been reported in the superlattice of CsPbBr_3_ quantum dots (QDs), exhibiting varying degrees of coherence depending on disorder and defects within the superlattice.^17^ Since the overlap of the exciton wave functions is crucial for creating a coherently coupled excited state, both the spatial proximity between the QDs and the quantum confinement of the QDs forming the superlattice should play an important role in producing superradiance. So far, cooperative photon emission from CsPbBr_3_ QDs was observed in 3D superlattices of weakly quantum-confined QDs passivated with relatively long surface ligands that limit the extent of spatial overlap of the exciton wave functions.^12,17,18^ Here, we report the polarized superradiance exhibiting a very narrow linewidth (<5 meV) and large spectral redshift (∼200 meV) at the excitation fluence as low as 5 nJ/cm^2^. This was achieved via a combination of strong quantum confinement imposed on the highly uniform ensemble of QDs and ligand engineering on the QD surface. This strategy not only enhanced the electronic coupling but also introduced unexpected anisotropy, despite the absence of strong intrinsic asymmetry in the QDs, enabling polarized superradiance, which is more suitable for applications in photonic devices than randomly polarized emission.

To investigate how the control of quantum confinement and spatial overlap of exciton wave functions alter the superradiant properties of a QD superlattice, CsPbBr_3_ QDs of two different sizes passivated with ligands of two different lengths were prepared. CsPbBr_3_ QDs of 9 nm and 4 nm with highly uniform size and shape, which are in a weak and strong quantum confinement regime, respectively (exciton Bohr radius: ∼3.5 nm), were synthesized as described in the Supporting Information. Oleylammonium bromide (OLAB) with 18 carbons and a bidentate ligand with 8 carbons (3C-C8) were used to vary the facet-to-facet distance between the QDs in the superlattice. Figure 1a–1d compare the solution-phase absorption and photoluminescence (PL) spectra of the QDs at room temperature that show the quantum confinement effect on exciton transition energy. Representative transmission electron microscopy (TEM) images of the QDs and the superlattice formed from each QD are shown in Figure 1e–1l. QDs passivated with OLAB and 3C-C8 provide facet-to-facet distances of 3–2.5 nm and ∼1.4 nm, respectively, in the close-packed assembly based on TEM image analysis. Details of superlattice preparation and structural characterization are provided in the Supporting Information (SI); see Figures S1–S3.

**Figure 1:**
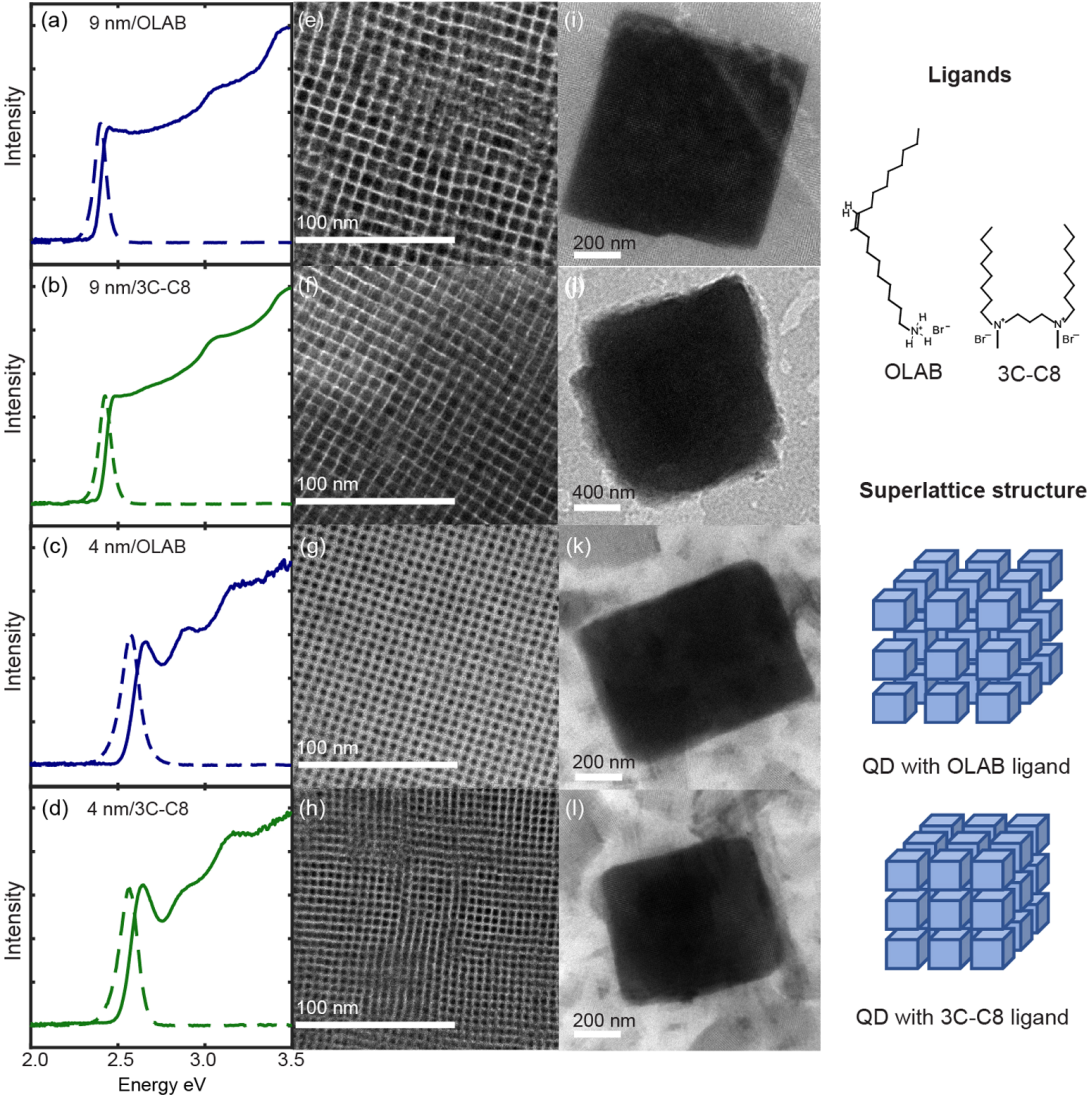
Optical spectra and TEM images of the size- and ligand-tuned CsPbBr_3_ quantum dots and superlattices. (a–d) Solution-phase absorption and photoluminescence spectra of CsPbBr_3_ QDs of different sizes and passivating ligands. Each panel is labeled with QD size/ligand: (a) 9 nm/OLAB, (b) 9 nm/3C-C8, (c) 4 nm/OLAB, and (d) 4 nm/3C-C8. (e–h) TEM images of a QD sample on a TEM grid; (i–l) TEM image of a superlattice formed from each QD sample. The right side of the figure shows the chemical structure of the OLAB and 3C-C8 ligands and an illustration of the superlattice structures with a ligand-tuned facet-to-facet distance between the QDs.

The blue curves in Figure 2a–2d are the PL spectra at 10 K measured from superlattices formed from the four different QDs shown in Figure 1. For comparison, the PL spectra from a dilute dispersion of the same QDs in a polystyrene matrix are shown in red. A 405 nm excitation at the fluence of 240 nJ/cm^2^ per pulse and the repetition rate of 5 MHz was used to ensure sufficiently low excitation density (0.03 and 0.003 per uncoupled QD for 9 and 4 nm QDs, respectively, based on the reported absorption cross section^19^). With OLAB ligand, 4 nm QDs show no noticeable sign of coupling between the QDs in their PL spectra, while 9 nm QDs show a small redshift indicative of some electronic coupling similar to what has been observed in a earlier study.^17^ With 3C-C8 ligands, on the other hand, both 4 and 9 nm QDs display an additional red-shifted peak with narrower linewidth. The reversible appearance and disappearance of this new peak upon temperature cycling between 10 and 300 K, along with a clear difference from the bulk-like CsPbBr_3_ nanocrystals in the temperature-dependent peak position and linewidth,^20,21^ rule out the merging of QDs in the superlattice. Figure S4 in the SI compares the different temperature-dependent peak shift and linewidth of the superradiance from this work and the bulk-like emission reported in ref (20), which supports the absence of merging of the QDs in our study. These new peaks are attributed to the superradiance of the coupled QDs in the superlattice. The higher-energy peaks are similar to those from the QDs dispersed in a polystyrene matrix, which are attributed to PL from a subpopulation of QDs not coupled in the superlattice.^12,22^ The 4 nm/3C-C8 QD superlattices typically exhibited the full width at half-maximum (fwhm) linewidth of 3–5 meV and a redshift of 180–220 meV relative to the uncoupled exciton PL (see Figure S5 in the SI). These are the narrowest linewidth and the largest redshift observed to date for cooperative photon emission from CsPbBr_3_ QDs as either superfluorescence or superradiance. The 9 nm/3C-C8 QD superlattice exhibited a 7–10 meV linewidth and ∼70 meV redshift. While the linewidth narrowing and the redshift of the superradiance are less pronounced than in the 4 nm/3C-C8 QD superlattice, these are comparable to those of superfluorescence recently reported for the superlattice made with OLAB-passivated QDs of similar size.^12,17^ If the linewidth and redshift are taken as the indicators of the extent of coupling between the QDs, the contrast in the PL spectra between the OLAB- and 3C-C8-passivated QDs highlights the importance of ligand tuning in obtaining the QD coupling necessary to produce superradiance.

**Figure 2:**
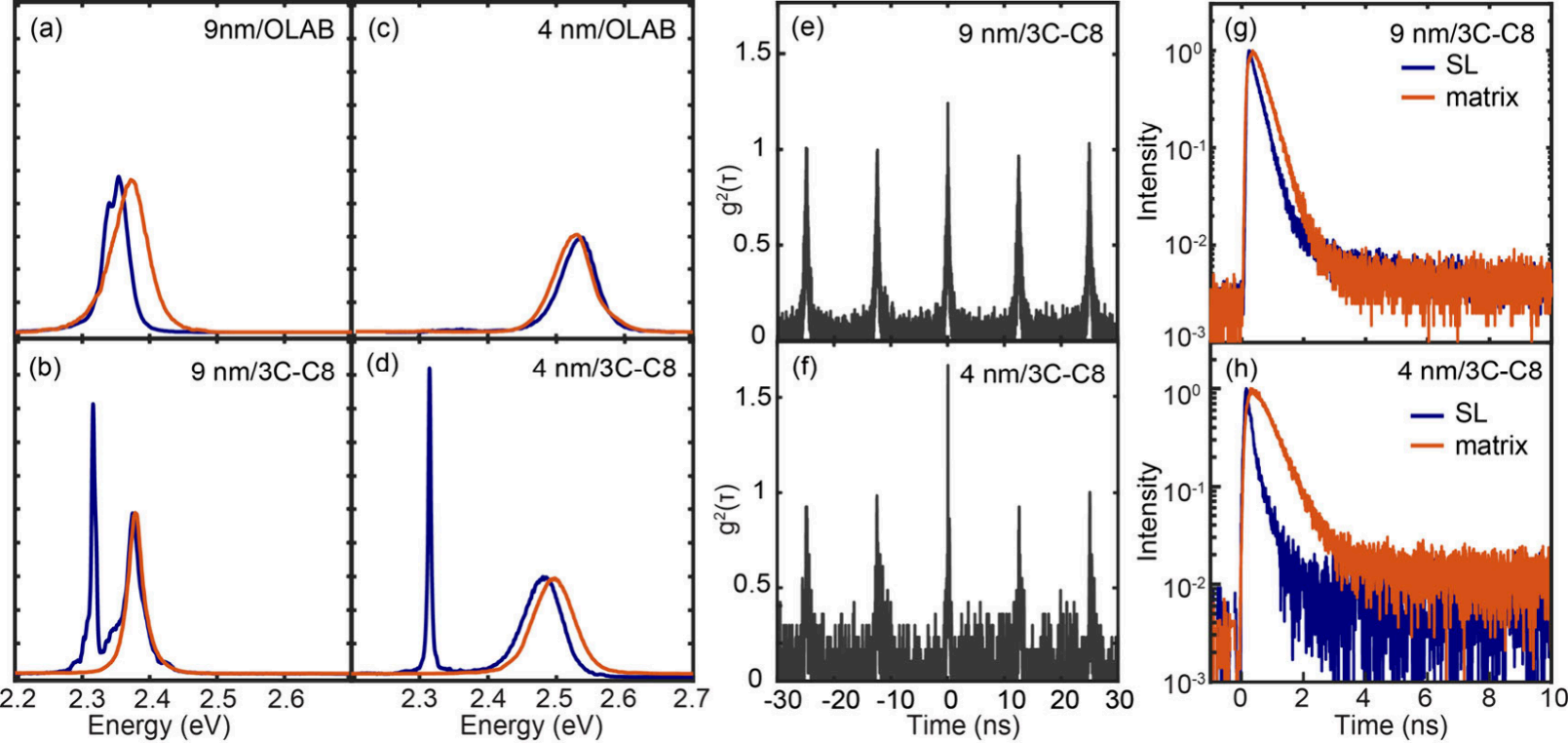
Emission spectra, second-order photon correlation, and lifetime of superradiance from superlattices. (a–d) PL spectra of the superlattice (blue) and dilute dispersion (red) of QDs measured at 10 K. The label inside each panel represents the QD size/ligand. (e, f) Second-order photon correlation, *g*^2^(τ), of superradiance from the superlattice formed from (e) 9 nm/3C-C8 and (f) 4 nm/3C-C8 QDs. (g, h) Comparison of the normalized time-dependent PL intensities of superradiance from the superlattice and diluted dispersion of QDs in the polymer matrix, (g) 9 nm/3C-C8 and (h) 4 nm/3C-C8 QDs.

The features of cooperative emission from coupled QDs include photon bunching and an accelerated radiative decay rate. Photon bunching was confirmed through second-order photon correlation, *g*^2^(τ), measurements using a Hanbury Brown-Twiss interferometer. Spontaneous emission from uncorrelated emitters shows the random Poisson distribution of photon arrival times, giving an average *g*^2^(τ) value of 1, while photons from correlated emitters tend to bunch with *g*^2^(τ) < *g*^2^(0) and *g*^2^(0) > 1.^23,24^Figure 2e and 2f show the *g*^2^(τ) profiles of superradiance from the superlattices formed from 9 nm/3C-C8 and 4 nm/3C-C8 QDs measured at 10 K, both of which exhibit photon bunching at τ = 0. For these measurements, a 405 nm picosecond pulsed laser at the repetition rate of 80 MHz and photon fluence of 240 nJ/cm^2^ was used. The 4 nm/3C-C8 QDs show *g*^2^(0) = 1.6, significantly larger than that of 9 nm/3C-C8 QDs. This indicates the higher cooperativity of the superradiance likely due to the larger spatial overlap of the exciton wave functions between more strongly confined QDs. In contrast to the superradiance, the exciton PL from uncoupled QDs shows a constant *g*^2^(τ) value at all delay times, as expected from random uncoupled emitters (see Figure S6 in the SI). Figure 2g and 2h compare the time-resolved intensities of the superradiance and PL from uncoupled QDs in the superlattice formed from 9 nm/3C-C8 and 4 nm/3C-C8 QDs, measured at 10 K. The 4 nm/3C-C8 QD superlattice shows 3-fold faster decay of superradiance (150 ps) than the PL from uncoupled QDs (490 ps) that exhibit the same decay rate of exciton PL from a dilute dispersion of QDs in a polymer matrix. This acceleration can be attributed to the increased strength of dipole via coherent coupling of the QDs, similarly to the findings by Blach et al.^17^ The acceleration of the decay of superradiance from the 9 nm/3C-C8 QDs superlattice is weaker, showing a 1.4-fold acceleration compared to the PL from uncoupled QDs (260 ps vs 380 ps), indicating the weaker coupling than in 4 nm/3C-C8 QDs.

Compared to the superfluorescence previously reported for weakly confined CsPbBr_3_ QDs passivated with a long ligand,^12,25^ the superradiance from the coupled QDs investigated here exhibits several differences. One is the linear excitation fluence dependence of both the peak and integrated intensities of superradiance, whereas the peak and integrated intensities of superfluorescence reported in ref (12) are superlinear and linear, respectively, to the excitation fluence due to the varying spectral linewidth (see Figure S7 in the SI). Figure 3a and 3b show the excitation fluence dependence of the peak and integrated superradiance intensity at 10 K, measured from the superlattice formed from 9 nm/3C-C8 and 4 nm/3C-C8 QDs, exhibiting a linear excitation intensity dependence. The spectral line shape of the superradiance from superlattices of both 9 nm/3C-C8 and 4 nm/3C-C8 QDs, which is independent of the excitation fluence, is shown in Figure S7a and b of the SI. The excitation fluence dependence of the linewidth from this study and ref (12) is also compared in Figure S7e in the SI. The decay time of the superradiance is also independent of the excitation fluence as shown in Figure 3e and 3f, in contrast to the superfluorescence, which exhibits faster decay with increasing excitation fluence.^12,15^ The comparison of the excitation fluence dependence of the decay times from this study and ref (12) is made in Figure S7c and d of the SI. The absence of excitation fluence dependence of superradiance discussed above indicates that the coupling of QDs is independent of the excitation fluence, unlike the previously reported superfluorescence, since the cooperative emission emerges directly from the coherently coupled emitting state.

**Figure 3:**
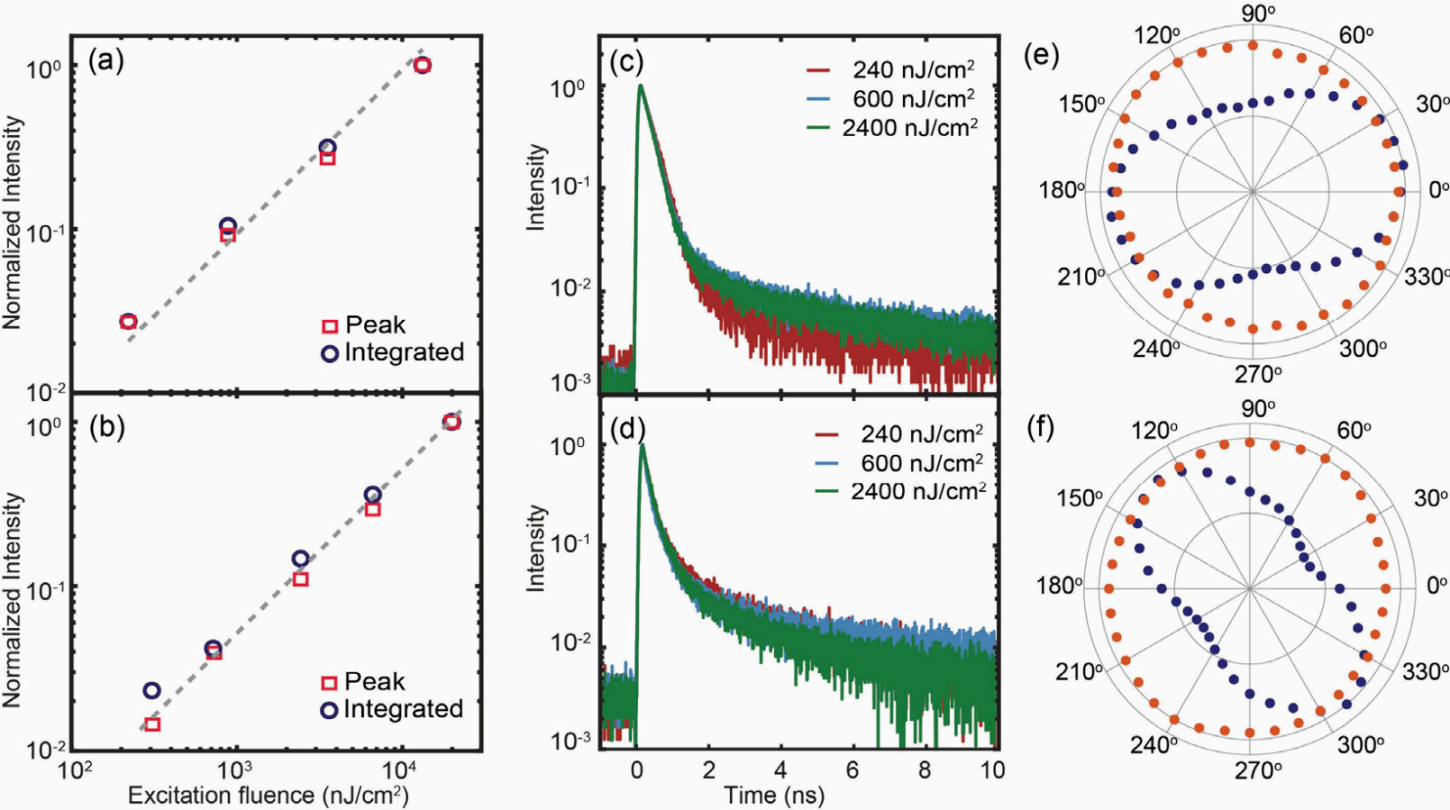
Excitation fluence dependence of superradiance and polarization anisotropy. (a, b) Excitation fluence dependence of peak (□) and integrated (○) superradiance intensity at 10 K. Dashed lines represent the linear excitation fluence dependence. (c, d) Normalized excitation fluence dependence of superradiance decay at 10 K. (e, f) PL polarization anisotropy of superradiance (blue) and uncoupled exciton PL (red) at 10 K. (a, c, e) Superlattice formed from 9 nm/3C-C8 QDs and (b, d, f) superlattice formed from 4 nm/3C-C8 QDs.

A unique and unexpected feature observed in the superradiance from this study that has not been previously observed is the polarization anisotropy of the emission. In the absence of intrinsic anisotropy in exciton transition in individual nanocrystals, such as nanoplatelets and nanorods, or the geometry of the superlattice that introduces anisotropy in the light propagation or interaction with QDs, a superlattice is not expected to exhibit the anisotropy of emission.^26,27^ Cube-shaped CsPbBr_3_ QDs do not possess a significant anisotropy of the exciton PL due to transition dipoles present along each axis.^28,29^ Therefore, the ordered array of the QDs within the superlattice should not exhibit polarization anisotropy of exciton PL at ambient temperature, as confirmed from a separate experiment (see Figure S8 in the SI). Figure 3e and 3f compare the polarization anisotropy of the superradiance and uncoupled exciton PL measured at 10 K from superlattices formed from 9 nm/3C-C8 and 4 nm/3C-C8 QDs. Superradiance from both 9 nm/3C-C8 and 4 nm/3C-C8 QD superlattices exhibits a preferred polarization direction, in contrast to the nearly isotropic uncoupled exciton PL. Since both superradiance and uncoupled exciton PL come from the same superlattice, the absence of polarization anisotropy in the uncoupled exciton PL rules out the possibility that the geometry of the superlattice introduces extrinsic anisotropy in the measured PL signal, such as the cavity effect reported earlier.^30^ The polarization angle-dependent superradiance is only in its intensity without any change in spectral shape or peak position (see Figure S9 in the SI). Therefore, this observation is interpreted as arising from the anisotropic electronic coupling of the QDs within the superlattice. The preferred polarization direction of the superradiance was independent of the polarization of excitation light at 405 nm that excites above the bandgap of the QDs, and the superradiance intensity did not exhibit dependence on excitation polarization. It is unclear how anisotropic electronic coupling could arise in the superlattice without an immediately identifiable cause that breaks the symmetry. Further studies exploring more detailed structure–polarization property relationships, including the polarization-dependent absorption measurement that is beyond our current instrumental capability, will shed more light on this interesting phenomenon. We conjecture that slight asymmetries in the QD structure and inter-QD interaction may significantly impact the formation of anisotropic coupled dipoles. Nevertheless, the potential to construct polarized superradiant light sources using superlattices fabricated from perovskite QDs could expand the applications of perovskite QDs as the source of quantum photons with controlled characteristics.

The necessary coherence between the QDs to produce superradiance generally decreases with increasing temperature.^31−33^ To examine the robustness of the coherence in the superlattice, temperature-dependent PL spectra and PL decay were measured. Figure 4a and b show the PL spectra in the temperature range 10–300 K measured from the superlattices of 9 nm/3C-C8 and 4 nm/3C-C8 QDs. The peak attributed to the superradiance begins to appear at 120 K from the 4 nm/3C-C8 QD superlattice, and its intensity increases with decreasing temperature. In the 9 nm/3C-C8 QD superlattice, the superradiance begins to appear at 30 K, significantly lower than in the 4 nm/3C-C8 QD superlattice. This suggests that the stronger quantum confinement of the QDs that allows the larger exciton wave function overlap helps maintains the coherence in the coupled QDs at higher temperatures. In Figure 4b, the redshift of superradiance with respect to the uncoupled exciton PL increases with decreasing temperature, consistent with the higher degree of coupling of QDs at the lower temperature. The small temperature-dependent shift of the exciton PL peak reflects primarily the variation of the bandgap with the temperature.^32,34^ Temperature-dependent decays of superradiance and uncoupled exciton PL are also compared in Figure 4c–4f. The 4 nm/3C-C8 QD superlattice exhibits significant acceleration of the decay of the superradiance with the decrease of temperature, in contrast to the uncoupled exciton PL, which shows substantially weaker temperature dependence. This is consistent with the interpretation of the acceleration of the radiative decay rate resulting from the increase of the dipole strength via coherent coupling of the QDs.^35^ The temperature dependence of the peak position and linewidth of both superradiance and uncoupled exciton PL are shown in the SI (see Figure S10).

**Figure 4:**
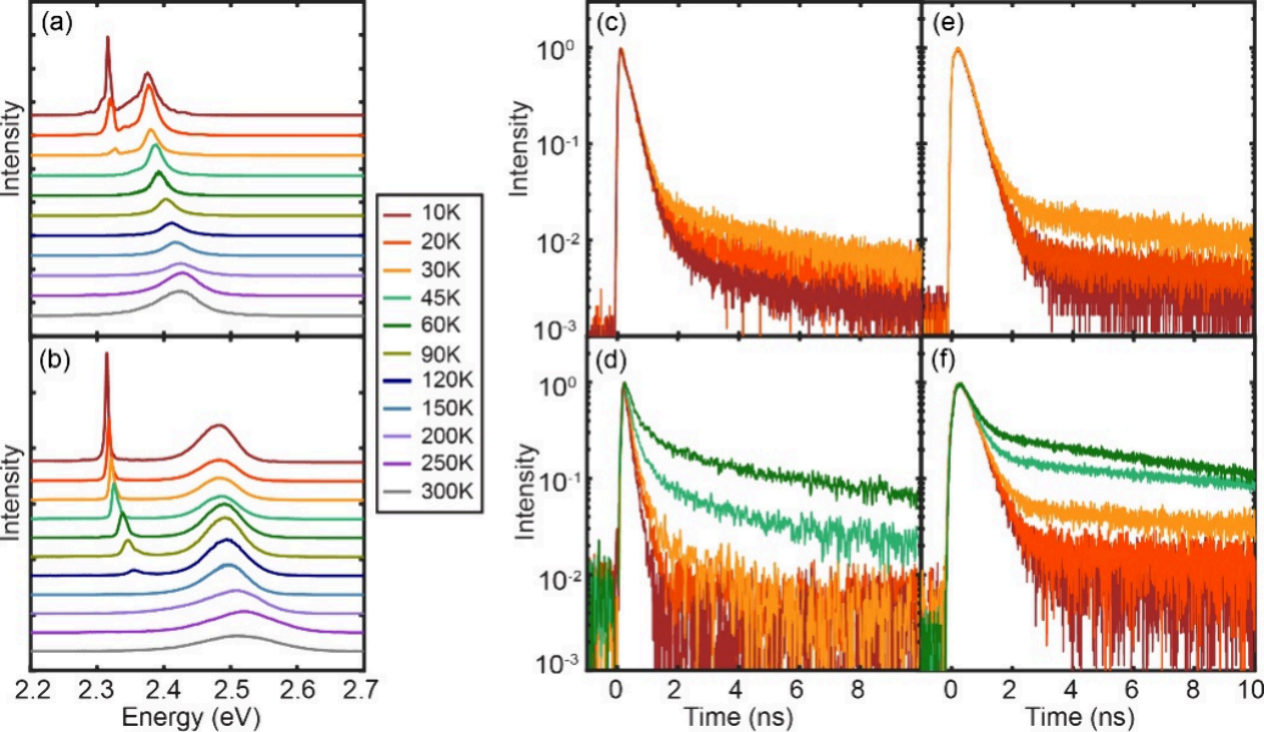
Temperature dependence of superradiance. (a, b) Temperature-dependent PL spectra of the superlattices formed from (a) 9 nm/3C-C8 and (b) 4 nm/3C-C8 QDs. (c, d) Temperature-dependent decay of superradiance from the superlattices formed from (c) 9 nm/3C-C8 and (d) 4 nm/3C-C8 QDs. (e, f) Temperature-dependent decay of uncoupled exciton PL from (e) 9 nm/3C-C8 and (f) 4 nm/3C-C8 QDs.

The main spectral features of superradiance, their temperature dependence, and the polarization anisotropy observed in this study can be well-formulated using a model described below, which reproduces results that are in qualitative agreement with those of the experiment. A more detailed description of the simulation is provided in the Supporting Information. We treat the uncoupled QDs as two-level quantum emitters with a Gaussian distribution of exciton transition energy centered at 2.485 eV with the fwhm linewidth of 52 meV, matching the position and linewidth of the observed broad peak in the PL spectrum between 2.4 and 2.6 eV. The electronic coupling between QDs is then introduced via the tight-binding Hamiltonian,
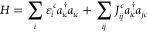
1where *a*_*ic*_, *a*_*ic*_^†^ are the annihilation and creation operators for the excited state of the *i*th exciton. For convenience, we assume that the ground state of all excitons is the same, whereas the excited state energies ε_*i*_^*c*^ differ. Taking into account only nearest-neighbor coupling, we can either numerically diagonalize the Hamiltonian to find eigenenergies for a given number of QDs or assume a large enough periodic superlattice, so that one can define momentum ***k*** = (*k*_*x*_, *k*_*y*_, *k*_*z*_) and go to the limit of the momentum-space Hamiltonian and continuum bands.^36^ The energy dispersion in this limit is ε_*c***k**_ = ε_0_ – 2*J*_*x*_ cos(*k*_*x*_*a*) – 2*J*_*y*_ cos(*k*_*y*_*a*) – 2*J*_*z*_ cos(*k*_*z*_*a*), which provides a good fit to the discrete energies at low momenta, which make the dominant contribution to the PL at low temperatures. Here, *a* is the superlattice period and *J*_*x*,*y*,*z*_ are the components of the hopping parameter, i.e., essentially the Fourier amplitudes of expanding the interaction Hamiltonian in eq 1 in the momentum space. In this model, the narrow PL peak from the coupled QDs is due to optically excited carriers that relax to the bottom of the lowest excited band at around *k* = 0 before their recombination. The observed redshift of this peak relative to the uncoupled PL spectrum is determined by the magnitudes of the hopping parameters. We fixed their values by comparing them with the observed spectra at 10 K. The hopping parameter decreases with increasing temperature due to vibrational (phonon) excitations, which are activated with probability e^–*E*_v_/(*k*_B_*T*)^, where *E*_v_ is the characteristic vibrational energy and *k*_B_ is the Boltzmann constant. Therefore, the hopping parameter scales with temperature as . Within this model, there is no qualitative difference between the PL from 2D and 3D superlattices other than the rescaling of the numerical values of the hopping parameters. Therefore, to save computation time, we used a 2D model with two components, *J*_*x*_ and *J*_*y*_, having different values to account for the observed anisotropy as explained below. At low temperatures, the width of the PL peak from the coupled QD state is dominated by the homogeneous linewidth γ(*T*) of the emission from a given *k*-state. Its value decreases linearly with decreasing temperature due to reduced electron–phonon scattering.^37^ The resulting calculated PL spectra are shown in Figure 5a, which are in good agreement with the data in Figure 4b for the 4 nm/3C-C8 QD superlattice. The difference between 4 nm/3C-C8 QD and 9 nm/3C-C8 QD superlattices can be explained by lower interband transition energy and smaller hopping parameters for the larger QDs and the difference in the phonon density of states. The anisotropy of superradiance cannot be explained by the shape of the superlattice or the intrinsic anisotropy of the individual QDs as mentioned earlier. Therefore, we assumed that it results from the anisotropy in the inter-QD coupling, i.e., unequal values of the hopping parameters *J*_*x*_ and *J*_*y*_ and resulting optical dipole matrix elements *d*_*x*,*y*_ ∝ *J*_*x*,*y*_ at small enough *J*, see eq 4 in the Supporting Information. The observed polarization anisotropy of superradiance shown in Figure 3f is best reproduced with *J*_*x*_/*J*_*y*_ ≈ 3/2, as shown in Figure 5b, ignoring the actual direction of the anisotropy axis in the superlattice.

**Figure 5:**
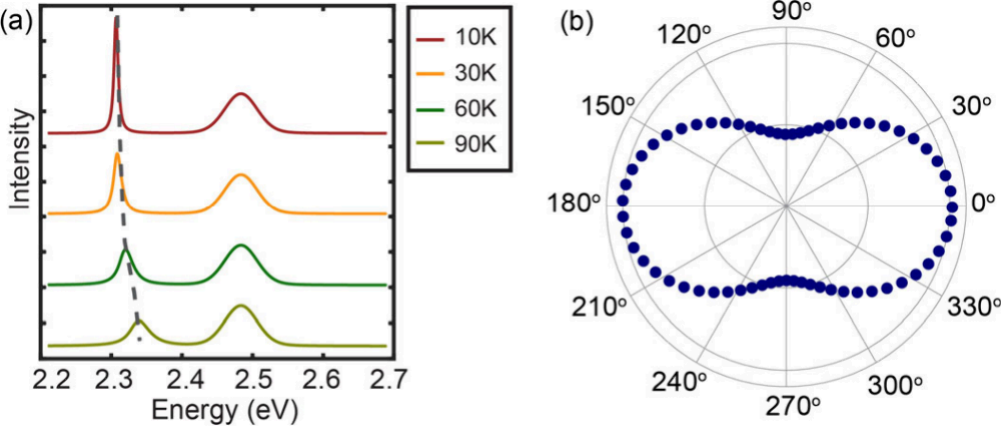
Simulated superradiance spectra from the tight-binding model. (a) Simulated superradiance spectra from the coupled QDs at different temperatures calculated using the best-fit parameters of the tight-binding model to the experimental data. The best-fit parameters are *E*_*v*_ = 0.015 eV, *J*_*x*_ = 0.06 eV, *J*_*y*_ = 0.04 eV, ε_0_ = 2.5 eV, and γ(*T* = 0) = 2 meV. The dashed gray curve shows the measured superradiance peak position. (b) Simulated polarization anisotropy of superradiance with respect to the horizontal *x*-axis for *d*_*x*_/*d*_*y*_ = 3/2. Other parameters are the same as in panel (a).

In conclusion, we report the observation of polarized superradiance from the electronically coupled CsPbBr_3_ perovskite QDs in the superlattice. Unlike superfluorescence emerging from the buildup of coherence from the incoherently excited emitters, the superradiance from the coupled QDs is governed by inter-QD electronic coupling that can be tuned by the quantum confinement and ligand engineering of the QDs forming the superlattice. We observed that the combination of strong quantum confinement and the use of the shorter ligand not only enhances the inter-QD coupling but also introduces anisotropy in the coupling, enabling the polarized cooperative photon emission. These results demonstrate the potential of the ordered assembly of perovskite QDs with controllable electronic coupling as the source of the polarized superradiant light.

## Supporting Information Available

Experiment details, including synthesis of QDs, SLs, isolated QD polystyrene matrix film, ligand synthesis, ligand exchange and steady state, time-resolved PL, second-order correlation measurements; supplemental figures (Figures S1–S11) including ^1^H NMR of 3C-C8 ligand, histogram of NC sizes obtained from TEM images, confocal images of SLs and isolated QD PL film, variations of superradiance among different superlattices, second-order correlations of uncoupled exciton in superlattices, excitation fluence dependent PL spectra of superlattices, polarization anisotropy of uncoupled exciton PL from the superlattices, polarization-dependent PL spectra of superlattice, temperature-dependent PL spectra of the dilute dispersion of CsPbBr_3_ QDs in polystyrene matrix, schematic diagram of the confocal microscope and spectroscopic measurement setup (PDF)
