# Supplementary material for: Polarized Superradiance from CsPbBr3 Quantum Dot Superlattice with Controlled Interdot Electronic Coupling
Source: Nano Lett. 2025 Apr 1;25(15):6176–83. doi: 10.1021/acs.nanolett.5c00478 (PMC12007084; doi:10.1021/acs.nanolett.5c00478)
Supplement: Supplementary file 1 — nl5c00478_si_001.pdf [file nl5c00478_si_001.pdf]

Polarized Superradiance from $CsPbBr_3$ Quantum Dot Superlattice with Controlled Inter-dot Electronic Coupling

Lanyin Luo[1,2], Xueting Tang[3], Junhee Park[3], Chih-Wei Wang[3], Mansoo Park[4], Mohit Khurana[1,2], Ashutosh Singh[1], Jinwoo Cheon[4], Alexey Belyanin[1], Alexei V. Sokolov[1,2] and Dong Hee Son[1,3,4]

[1]Department of Physics and Astronomy, Texas A&M University, College Station, TX 77843, USA
[2]Institute for Quantum Science and Engineering, Texas A&M University, College Station, TX 77843, USA
[3]Department of Chemistry, Texas A&M University, College Station, Texas 77843, United States
[4]Center for Nanomedicine, Institute for Basic Science and Graduate Program of Nano Biomedical Engineering, Advanced Science Institute, Yonsei University, Seoul 03722, Republic of Korea

[*]Corresponding author E-mail: dhson@chem.tamu.edu

Supplementary Notes

1. Synthesis of $CsPbBr_3$ quantum dots (QDs) and superlattice fabrication

Materials. Cesium carbonate (99.994%, Alfa Aesar), lead bromide (98%, Alfa Aesar), zinc bromide (99%, BeanTown Chemical), hydrobromic acid (48%, VWR), 1-octadecene (90% technical grade, Sigma-Aldrich), oleylamine (≥98%, Sigma Aldrich), oleic acid (90% technical grade, Sigma-Aldrich), 1,3-dibromopropane (98% TCI), *N*,*N*-dimethyloctylamine (97% Sigma-Aldrich), *N*,*N*-dimethyldodecylamine (96% TCI), were used as received without further purification.

Synthesis of 9 nm $CsPbBr_3$ QDs passivated with oleylammonium bromide (OLAB). The synthesis procedure was adopted from a report by Yakunin et al.[1] Cesium oleate was prepared by combining Cs_2CO_3 (250 mg), oleic acid (OA, 0.8 g) and 1-octadecene (ODE, 7 g) in a 50 ml three-neck round-bottomed flask under a nitrogen atmosphere. The flask was evacuated at room temperature for 10 minutes before being heated to 150 °C for an additional 10 minutes. The flask was refilled with nitrogen and held at 120 °C for further use. In another 50 mL round-bottomed flask, $PbBr_2$ (140 mg), OA (1 mL), oleylamine (OAm, 1 mL), and ODE (10 mL) were combined under nitrogen and stirred at room temperature for 10 minutes. The flask was then evacuated for 20 minutes before being refilled with nitrogen. It was heated to 120 °C until the precursors fully dissolved and heated further to 220 °C. To initiate the reaction, 0.8 mL of Cs-oleate was swiftly injected into the flask. The reaction was allowed to proceed for 5 seconds before quenching with an ice bath. To purify the resulting QDs, the crude solution was centrifuged to precipitate the QDs and the recovered QDs were redisperse in hexane. The QDs were precipitated again by adding methyl acetate to the hexane solution, and the recovered QDs were resuspended in hexane for further use.

Synthesis of 4 nm $CsPbBr_3$ QDs passivated with OLAB. The synthesis procedure was adopted from a report by Dong et al.[2] Cesium oleate was prepared by combining Cs_2CO_3 (600 mg), OA (2.4 mL) and ODE (6.4 mL) in a 50 ml three-neck round-bottomed flask under a nitrogen atmosphere. The flask was evacuated at room temperature for 15 minutes before being heated to 120 °C for an additional 15 minutes. The flask was refilled with nitrogen and held at 120 °C for further use. In a separate 100 mL round-bottomed flask, $PbBr_2$ (350 mg), $ZnBr_2$ (700 mg), OA (7 mL), OAm (7 mL) and ODE (20 mL) were mixed under nitrogen and stirred at room temperature for 10 minutes. The flask was then evacuated for 20 minutes before being refilled with nitrogen. The flask was heated to 150 °C for 20 minutes and cooled to 100 °C. To initiate the reaction, 3 mL of Cs-oleate was swiftly injected into the flask. The reaction was allowed to proceed for an hour before being quenched with an ice bath. To purify the resulting

QDs, acetone was added to the crude solution to precipitate the QDs. Supernatant was separated and discarded via centrifugation, and the precipitate was resuspended in hexane for further use.

Synthesis of 3C-C8 ligands. The 3C-C8 ligand was synthesized using the method adapted from a previous report.[3] A bidentate ligand was chosen because it was effective in providing the stability of the QDs and superlattice when short-chain ligand is used.[3, 4] Tertiary amine (8 mmol) and 1,3-dibromopropane (0.404 g, 2 mmol) were dissolved in 5 mL acetonitrile in a 25 mL round-bottomed flask. A condenser was connected to the flask and the flask was purged with nitrogen. The mixture was refluxed overnight under a nitrogen atmosphere, then cooled to room temperature. The crude product was precipitated by centrifugation after adding diethyl ether. Additional diethyl ether was added to the precipitate and sonicated for 15 minutes before isolating the product by centrifugation. The resulting white powder was dried in vacuo overnight. Recrystallization with toluene was performed to purify the ligands before use. The identity and purity of the product were confirmed by nuclear magnetic resonance (NMR) spectroscopy. NMR spectrum is shown in Figure S1.

Ligand exchange of OLAB-passivated $CsPbBr_3$ QDs with 3C-C8. All $CsPbBr_3$ QDs passivated with 3C-C8 ligand were prepared via ligand exchange of the OLAB-passivated $CsPbBr_3$ QDs. 250 μL of concentrated OLAB-passivated $CsPbBr_3$ QD solution ($\geq$ 0.25 mM) was transferred to a centrifuge tube. Methyl acetate was then added to the solution to bring the total volume to 2 mL, partially stripping the native ligand from the QDs. The QDs were isolated from the solution via centrifugation. The precipitated QDs were redispersed in 100 μL of a toluene solution containing 3C-C8. The solution was vigorously mixed for 5 minutes. Methyl acetate was again added to bring the total volume to 2 mL, followed by centrifugation to obtain the pellet. The pellet was redispersed in toluene until it dissolved completely. The solution was centrifuged at 6000 rpm for 3 minutes, and the supernatant was collected. The exchange process was repeated three times to complete the exchange.

Fabrication of superlattice of $CsPbBr_3$ QDs. The superlattices of $CsPbBr_3$ QDs were fabricated via slow solvent evaporation following a previously reported procedure with modifications.[5] A silicon wafer used as a substrate (1cm ×1cm) was cleaned with sonication in a mixture of acetone and isopropyl alcohol. To remove the residual solvent and moisture, the substrate was dried with nitrogen gas followed by further drying in an oven. To form the superlattice, 40 μL of the diluted QD solution in toluene was dropped onto the silicon wafer and sealed in a PTFE well covered with a glass slide. The well was then transferred into a desiccator purged with nitrogen, where the self-assembly of the QDs was allowed to continue for 36 hours to form μm-sized cuboidal superlattices. The superlattices formed on the silicon wafer were used for all the optical measurements. The superlattices grown on TEM grids

employing the same method were used for imaging with a transmission electron microscope.

Fabrication of dilute dispersion of QDs in polystyrene matrix. To fabricate the isolated QDs dispersed in polystyrene matrix, a dilute QD solution was prepared using 6% polystyrene (MW 92,000) solution in toluene. The resulting optical density in 1 cm cuvette was ~1 at the exciton absorption peak. The diluted solution was directly drop-cast on a 1cm×1cm silicon wafer. The solvent was then removed in a vacuum oven, forming a uniform thin film of QDs dispersed in the polymer matrix.

Transmission electron microscopy of QDs. QD specimens were prepared by dropping 2.0 μL of concentrated QD solution onto carbon-supported copper grids (Ted Pella, 01840-F) pretreated with oxygen plasma (SolarusII, Gatan Inc., 20 W for 15 s) and drying under argon atmosphere. TEM images were acquired using a JEM 2100Plus (JEOL) electron microscope equipped with a scintillator-based camera (Gatan, OneView) and a specimen double-tilting holder (JEOL, EM-31630), operating at an acceleration voltage of 200 kV.

2. Spectroscopic characterization

Solution-phase absorption and PL spectra. Solution-phase absorption spectra of the QDs were obtained with a CCD spectrometer (Ocean Optics, USB2000) equipped with a deuterium light source (UV-VIS ISS, Ocean Optics). Steady-state PL spectra of the QD solutions were obtained with a CCD spectrometer (Ocean Optics, USB2000) and a 365 nm UV light emitting diode as an excitation source.

Steady-state PL spectra of the superlattice and dilute dispersion of QDs. Steady-state PL spectra of superlattice and dilute dispersion of QDs in polymer matrix were measured using a home-built confocal microscope equipped with a dual grating spectrograph (Princeton Instruments, SP-2150i) and a CCD camera (Princeton Instruments, PIXIS 100). (See Figure S10) A sample prepared on a silicon substrate was placed inside an open-cycle helium optical cryostat (JanisST-500), where the temperature was controlled between 10-300 K with a temperature controller (Lakeshore, Temperature Controller 332). A 405 nm diode laser (PicoQuant, P-C-405) was used as an excitation source. The beam size of our excitation light in our confocal microscope at the sample position is 0.6 μm.

Time-correlated single photon counting measurements. Time-resolved PL intensity of the superlattice and dilute dispersion of QDs in polymer matrix were measured using a time-correlated single photon counting setup that consists of a time correlation module (PicoQuant,

PicoHarp 300) and an avalanche photodiode (PicoQuant, MPD, PDM series). A 405 nm pulsed diode laser (PicoQuant, P-C-405) producing pulses of 45 ps width was used as the excitation source at a repetition rate of 5 MHz.

Second-order photon correlation measurement. Hanbury Brown-Twiss (HBT) interferometer setup was constructed for the measurement of second-order photon correlation. Two identical avalanche photodiodes (PicoQuant, MPD, PDM series) were used to detect the photons on each arm of the interferometer. A 405 nm pulsed diode laser (PicoQuant, P-C-405) producing pulses of 45 ps width was used as the excitation source at a repetition rate of 80 MHz.

PL and excitation polarized-dependent measurements. For the measurement of PL polarization anisotropy, a linear polarizer (Thorlabs, LPVISA100) was placed in front of the avalanche photodiodes and spectrometer. For the measurement of the excitation polarization-dependent PL spectra, the combination of a polarizing beamsplitter cube (Thorlabs, PBS251) and a half waveplate (Thorlabs, WPH10M-405) was used to vary the polarization of 405 nm excitation light.

3. Simulation of the temperature-dependent superradiance spectra

We treat the uncoupled QDs as two-level quantum emitters with a Gaussian distribution of exciton transition energies centered at 2.485 eV with the FWHM linewidth of 52 meV, matching the position and linewidth of the observed broad peak in the PL spectrum between 2.4-2.6 eV. Their PL is calculated by summing up the contribution of all emitters and assuming that each of them has a Lorentzian emission spectrum with a certain homogeneous linewidth; the value of the latter is not important as long as it is much smaller than the spread of transition energies.

Electronic coupling between QDs is introduced via the tight-binding Hamiltonian,

$$H = \sum_{i} \varepsilon_i^c a_{ic}^\dagger a_{ic} + \sum_{ij} J_{ij}^c a_{ic}^\dagger a_{jc} \tag{1}$$

where $a_{ic}, a_{ic}^\dagger$ are the annihilation and creation operators for the excited state of the ith exciton. For convenience, we assume that the ground state of all excitons is the same whereas the excited state energies ε_i^c differ. Taking into account only nearest-neighbor coupling, we numerically diagonalize the Hamiltonian to find eigenenergies for a given number of QDs. Assuming a large enough periodic superlattice, one can define momentum $\boldsymbol{k} = (k_x, k_y, k_z)$ and go to the limit of the momentum-space Hamiltonian and continuum bands.[6] The energy dispersion in this limit is

$$\varepsilon_{c\boldsymbol{k}} = \varepsilon_0 - 2J_x cos(k_x a) - 2J_y cos(k_y a) - 2J_z cos(k_z a), \tag{2}$$

where the parameters can be found by fitting the numerical results to the observed PL spectra. Here, a is the superlattice period and $J_{x,y,z}$ are the components of the hopping parameter, i.e., essentially the Fourier amplitudes of expanding the interaction Hamiltonian in Eq. (1) in the momentum space. In this model, the narrow and redshifted PL peak from the coupled QDs is due to optically excited carriers that relax to the bottom of the lowest excited band around $k = 0$ before their recombination. The fit of the energy bands from exact diagonalization of the Hamiltonian (1) with the continuum limit of Eq. (2) is not very good at high momenta. However, at our excitation fluences only about 10^{-3} of the QDs are excited, and at low temperatures only the states near $k = 0$ contribute to the PL.

We calculate the PL of the coupled QDs by summing up over the emission from thermally distributed excitations from k-states in the lowest tight-binding band with dispersion given by Eq. (2). Within this model, there is no qualitative difference between the PL from 2D and 3D superlattices, other than the rescaling of the numerical values of the hopping parameters. Therefore, to save computation time, we used a 2D model with two components J_x and J_y having different values to account for the observed anisotropy as explained below.

The observed redshift of the PL from coupled QDs relative to the uncoupled PL spectrum is determined by the position of the lowest tight-binding band, which, in turn, is controlled by the magnitudes of the hopping parameters. We determine these magnitudes from the observed spectra at the lowest temperature of 10 K. The hopping parameters decrease with increasing temperature due to vibrational (phonon) excitations, which are activated with probability $e^{-E_v/(k_B T)}$, where E_v is the characteristic vibrational energy and k_B is the Boltzmann constant. Therefore, the hopping parameters scale with temperature as $J_{x,y}(T) = J_{x,y}(0)\left(1 - e^{-\frac{E_v}{k_B T}}\right)$. The agreement with experiment showing decreasing redshift with increasing temperature is obtained for the value of E_v= 15 meV, which seems reasonable.

Furthermore, we take into account that the emission from each k-state is homogeneously broadened with the homogeneous linewidth $\gamma(T)$ at a given temperature T. At low temperatures of our experiment, the inhomogeneous broadening due to band dispersion is insignificant and the width of the PL peak from the coupled QD state is determined by the homogeneous linewidth $\gamma(T)$ at temperature T. Its value decreases linearly with decreasing temperature due to reduced electron-phonon scattering.[7]

The resulting PL spectra including the contribution of both uncoupled and coupled QDs and fitted with the experimental data for the 4 nm/3C-C8 QD superlattice are shown in Figure. 5a, in good agreement with the data in Figure. 4b. The difference between 4 nm/3C-C8 QD and 9 nm/3C-C8 QD superlattices can be explained by lower inter-band transition energy and smaller hopping parameters for the larger QDs and difference in phonon density of states.

The anisotropy of superradiance cannot be explained by the geometric shape of the sample or the intrinsic anisotropy of the individual QDs as mentioned earlier. Therefore, we assumed that it results from the anisotropy in the inter-QD coupling, i.e., unequal values of the hopping parameters J_x and J_y and resulting optical dipole matrix elements. As an example, we consider a class of tight-binding models which has a flat valence band at zero energy and a dispersive conduction band, corresponding well to the situation with coupled excitons in our QD superlattices. We modified their Hamiltonian to include anisotropic hopping parameters,

$$H_{\boldsymbol{k}} = \begin{pmatrix} 1 - 2J_y cos(k_y) & (1 - J_x e^{-ik_x})(-1 + J_y e^{-ik_y}) \\ (1 - J_x e^{ik_x})(-1 + J_y e^{ik_y}) & 1 - 2J_x cos(k_x) \end{pmatrix} \quad (3)$$

Here $k_{x,y}$ are normalized by the superlattice period, and all energies and $J_{x,y}$ are normalized by a certain energy scale. Proceeding in a standard way one can calculate the dipole matrix elements for the interband transitions via the dipole matrix elements of the momentum derivative of the Hamiltonian.[8] The resulting expressions are very cumbersome, but the $(k_x, k_y) \to 0$ limit is quite simple:

$$r_{x,y}^{(k_x,k_y)\to 0} = -\frac{iJ_{x,y}(J_{y,x}-1)}{2\sqrt{J_x^2(J_y^2-2J_y+2)-2J_x(J_y^2-J_y+1)+2J_y^2-2J_y+1}} \quad (4)$$

Here the components of the dipole matrix element are normalized by the superlattice period and electron charge. As one can see, $|r_{x,y}^{(k_x,k_y)\to 0}| \propto J_{x,y}$ for small $J_{x,y}$. The observed polarization anisotropy of superradiance shown in Figure 3f of the manuscript is best reproduced with $J_x/J_y \approx 3/2$ as shown in Figure 5b in the manuscript, ignoring the actual direction of the anisotropy axis in the superlattice.

Supplementary Figures

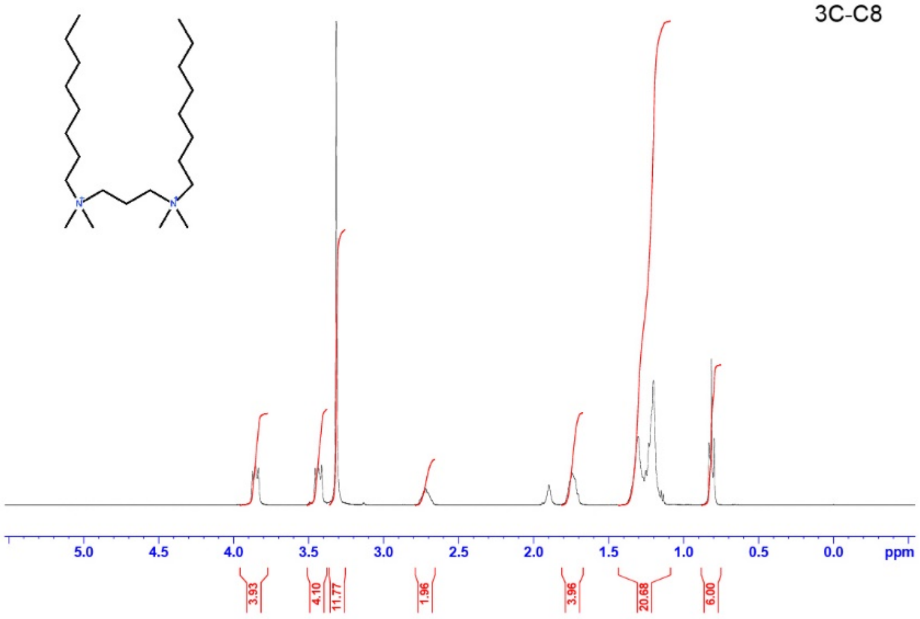

Fig. S1. ^{1}H NMR of 3C-C8 ligand synthesized. **3C-C8**: ^{1}H NMR (400MHz, $CDCl_3$): 3.87-3.83 (t, J = 7.8 Hz, 4H), 3.80-3.50 (m, 4H), 3.32 (s, 12H), 2.78-2.66 (m, 2H), 1.81-1.67 (m, 4H), 1.73-0.99 (m, 20H), 0.83-0.79 (t, J = 5.5 Hz, *6H)*

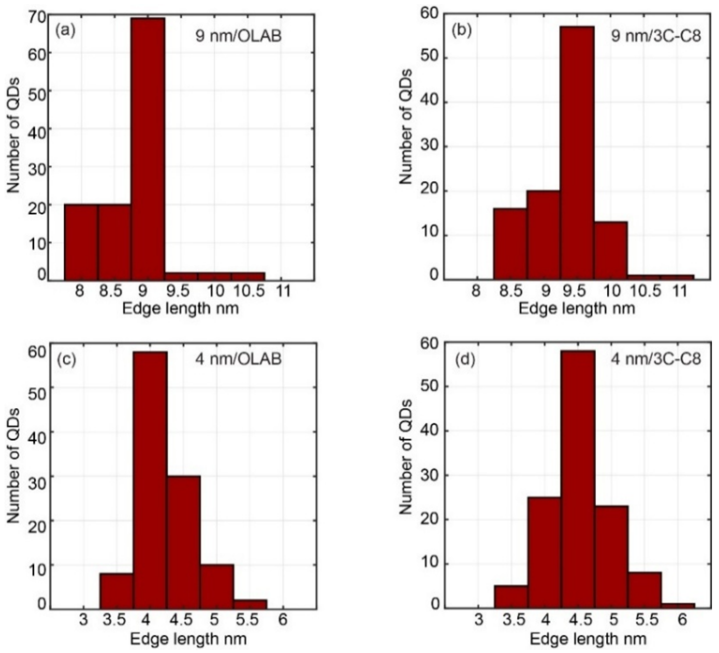

Fig. S2. Histogram of the NC sizes obtained from the TEM images in Fig. 1. (a) 9 nm QDs passivated with OLAB, (b) 9 nm QDs passivated with 3C-C8, (c) 4 nm QDs passivated with OLAB, (d) 4 nm QDs passivated with 3C-C8.

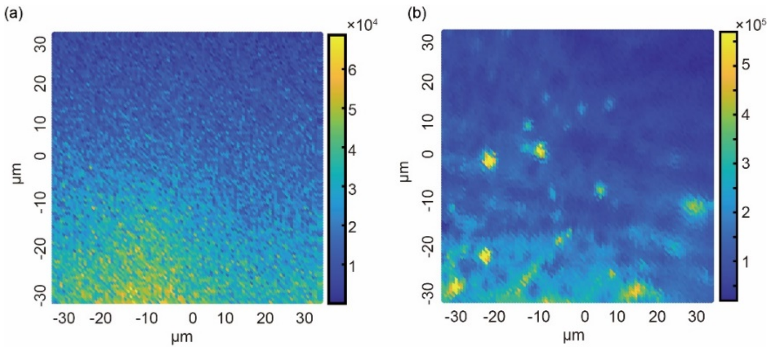

Fig. S3. Representative confocal fluorescence image. (a) dilute dispersion of $CsPbBr_3$ QDs in polystyrene matrix and (b) $CsPbBr_3$ QD superlattices obtained at 10 K.

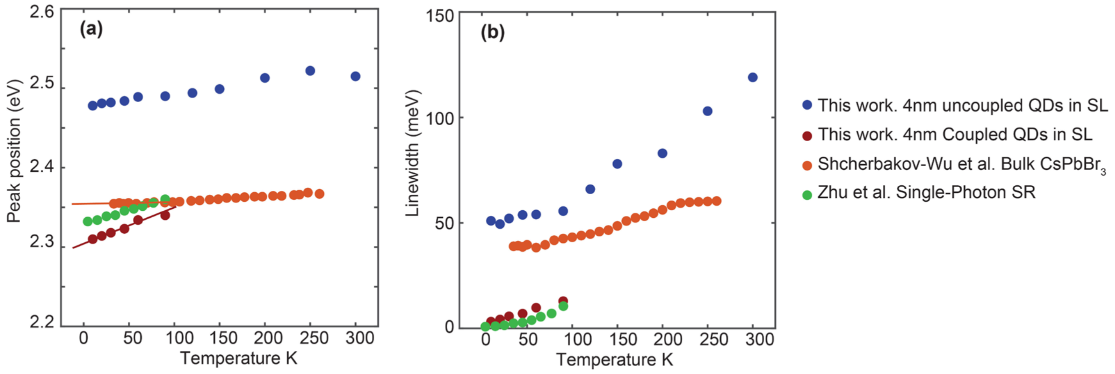

Fig. S4. Comparison of the temperature dependent-peak position (a) and linewidth (b) between the superradiance from this work and bulk-like emission from the literature (Shcherbakov-Wu et al.) The data reported for single-photon superradiance by Zhu et al (*Nature,* **626**, 535–541 (2024)) are also added for comparison.[4, 9]

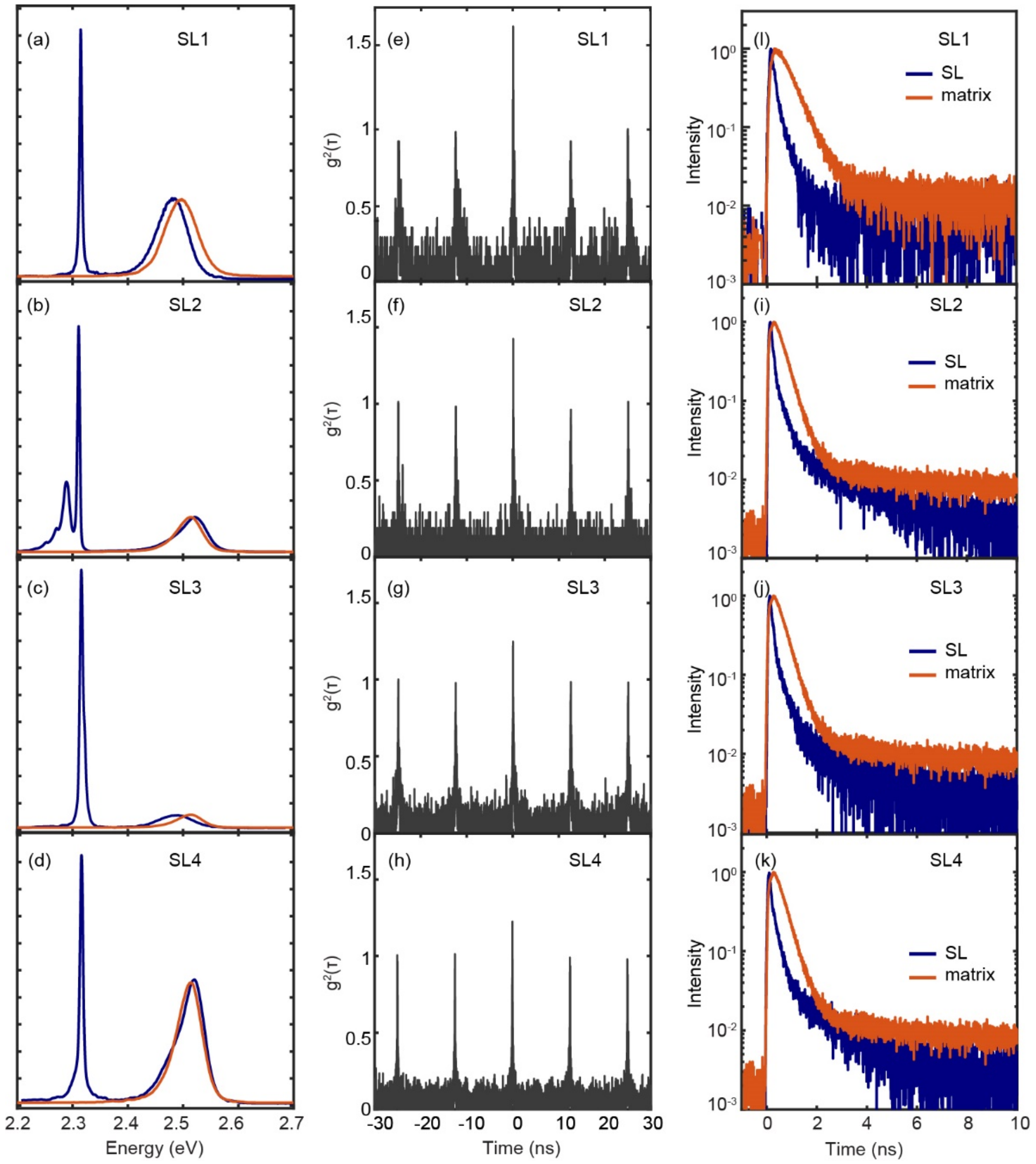

Fig. S5. Variations of superradiance among four different superlattices (SL1-SL4) formed from 4 nm/3C-C8 $CsPbBr_3$ QDs. (a-d) PL spectra of superlattices (blue) and dilute dispersion (red) at 10 K. (e-h) Second-order photon correlation, $g^2(\tau)$, of superradiance from superlattice. (l-k) Comparison of the normalized time-dependent PL intensities from superradiance and PL from uncoupled QDs. All data are obtained at 10 K under 405 nm pulsed excitation at the fluence of 240 nJ/cm^2. FWHM of superradiance varies in 3-5 meV range. The redshift of superradiance from the uncoupled exciton PL varies in 180-220 meV range. Significant photon bunching with $g^2(0) > 1.2$, and accelerated lifetime were detected, indicating cooperative emission from 4 nm/3C-C8 superlattice.

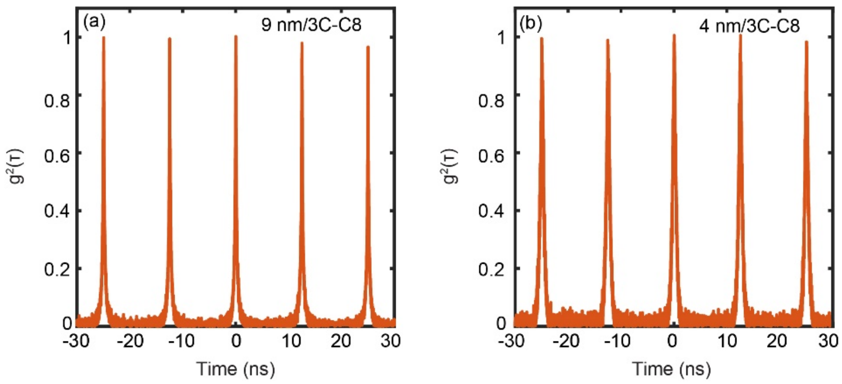

Fig. S6. Second order photon correlation, $g^2(\tau)$, of uncoupled exciton PL from the superlattice formed from (a) 9 nm/3C-C8 QDs and (b) 4 nm/3C-C8 QDs measured at 10 K.

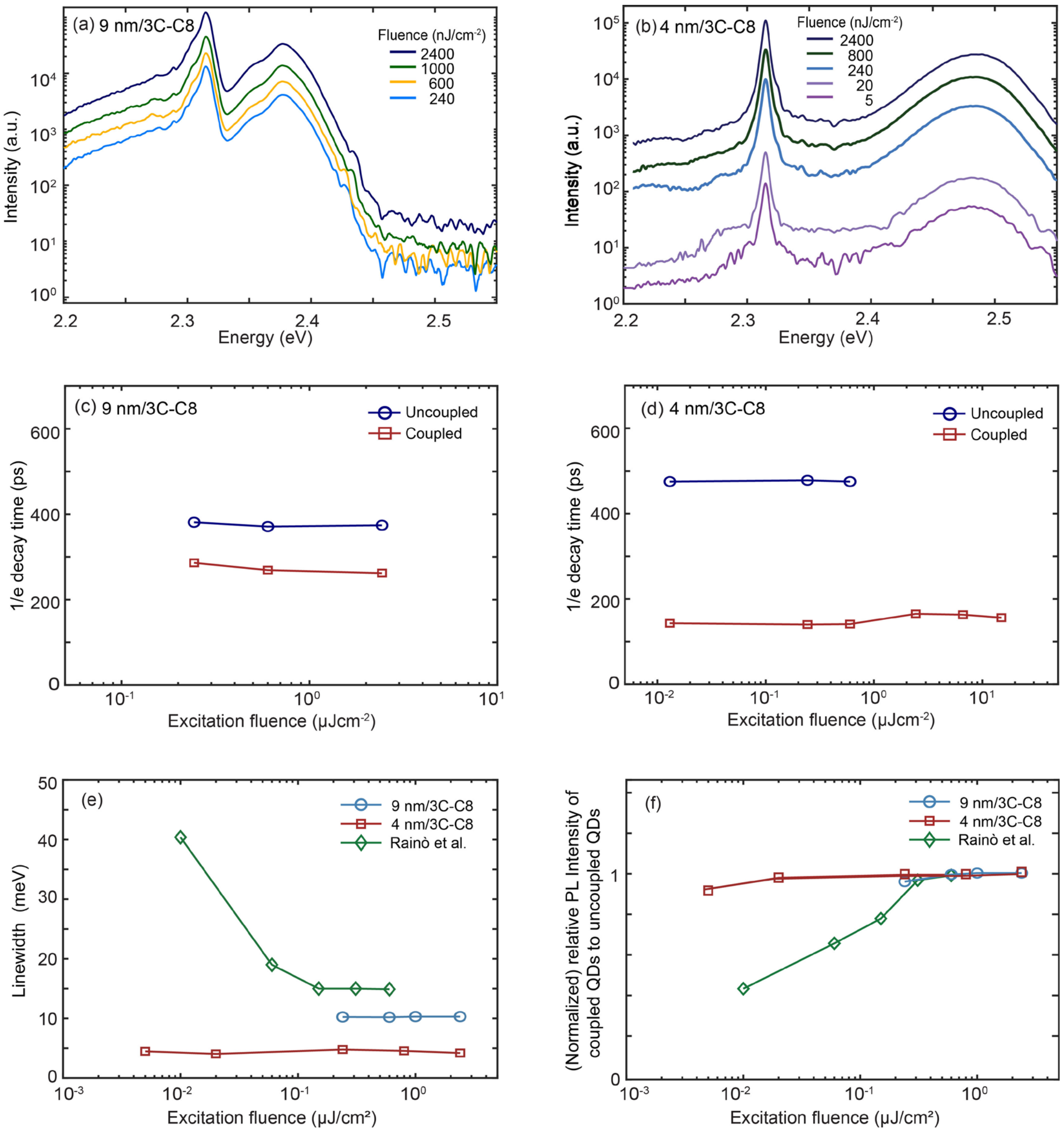

Fig. S7. Excitation fluence dependence at 10 K. (a) Spectra of 9 nm/3C-C8 QDs superlattice, (b) Spectra of 4 nm/3C-C8 QDs superlattice, (c) Decay times of the PL from coupled and uncoupled QDs in 9 nm/3C-C8 QDs superlattice, (d) PL decay times of from coupled and uncoupled QDs from 4 nm/3C-C8 QDs superlattice, (e) Comparison of the linewidths of the PL from the coupled QDs in the superlattice from this work and from Rainò work. (f) Comparison of the relative intensity of the PL from the coupled QDs relative to uncouple QDs in the superlattice from this work and from Rainò work. The relative intensity values are normalized to the value at the highest excitation fluence for an easy of comparison of the excitation fluence dependence.

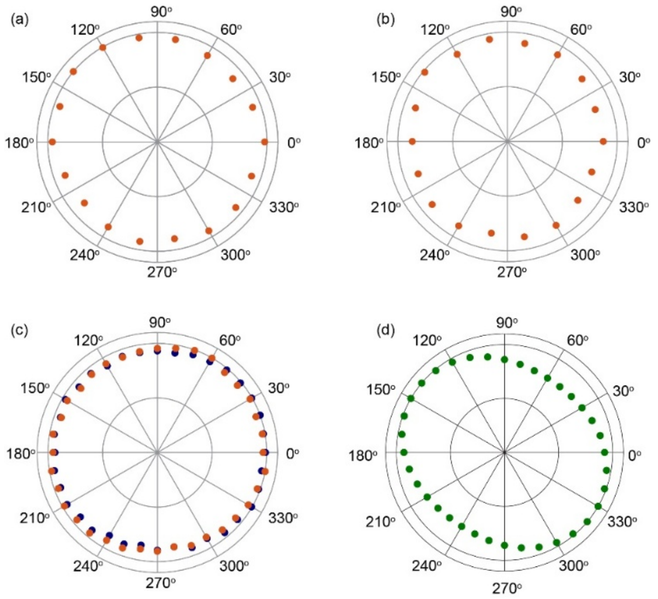

Fig. S8. Polarization anisotropy. All the polarization anisotropy plots are corrected for the extrinsic anisotropy introduced by the optical elements present between the sample and detector, including dichroic mirror and grating using the emission from randomly dispersed QDs in polymer matrix. (a, b) Polarization anisotropy of uncoupled exciton PL from the superlattices formed from (a) 9 nm/3C-C8 QDs and (b) 4 nm/3C-C8 QDs under 405 nm excitation at ambient temperature. (c) Excitation polarization-dependent intensity of superradiance (blue) and uncoupled exciton PL (red) both with 405 nm excitation from the superlattice formed from 4 nm/3C-C8 QDs at 10 K. (d) Uncorrected polarization dependence of emission from randomly dispersed QDs used to correct for the polarization anisotropy data reported in this study.

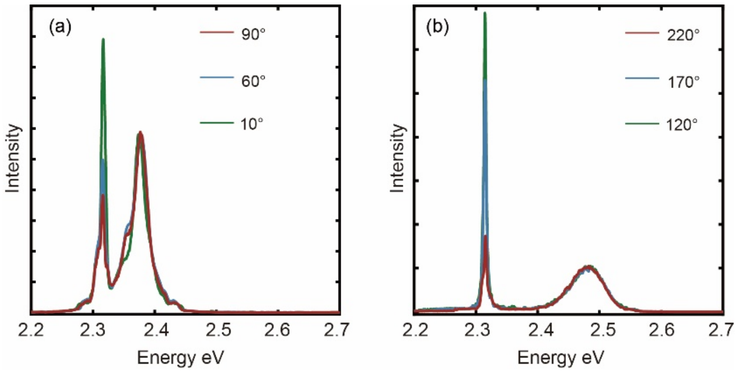

Fig. S9. Polarization-dependent PL spectra of superlattice formed from (a) 9 nm/3C-C8 QDs and (b) 4 nm/3C-C8 QDs at 10 K.

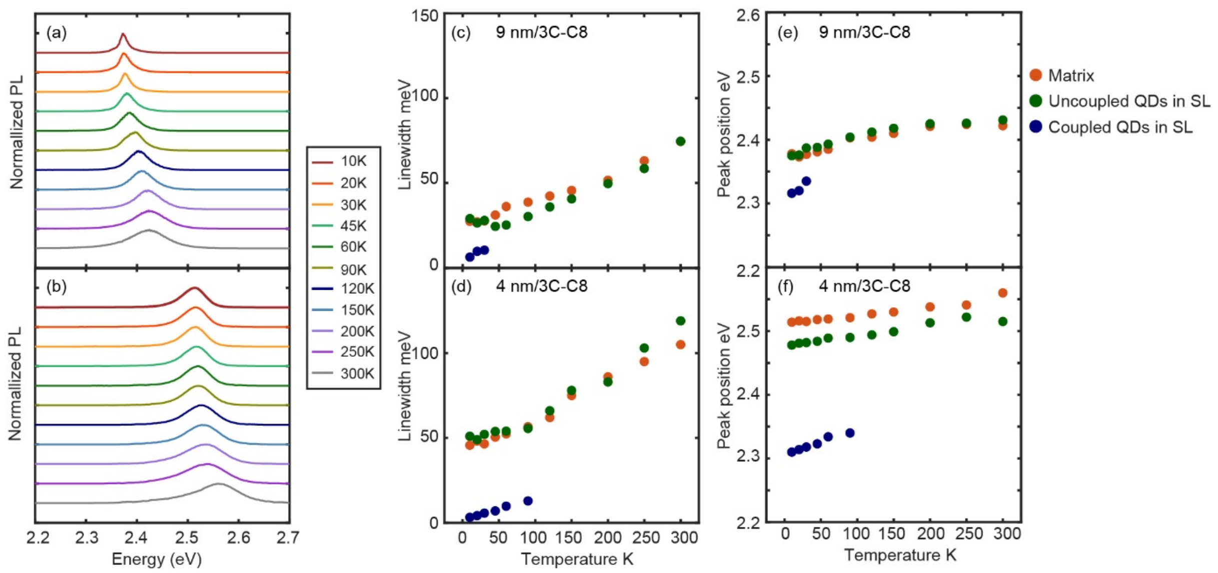

Fig. S10. (a, b) Temperature-dependent PL spectra of the dilute dispersion of $CsPbBr_3$ QDs in polystyrene matrix, (a) 9 nm/3C-C8, (b) 4 nm/3C-C8. (c, d) Temperature-dependent FWHM linewidth of superradiance and uncoupled exciton PL from superlattice and PL from dilute dispersion of QDs in polymer matrix, (c) 9 nm/3C-C8, (d) 4 nm/3C-C8. (e, f) Temperature-dependent peak position of superradiance and uncoupled exciton PL from superlattice and PL from dilute dispersion of QDs in polymer matrix, (e) 9 nm/3C-C8, (f) 4 nm/3C-C8.

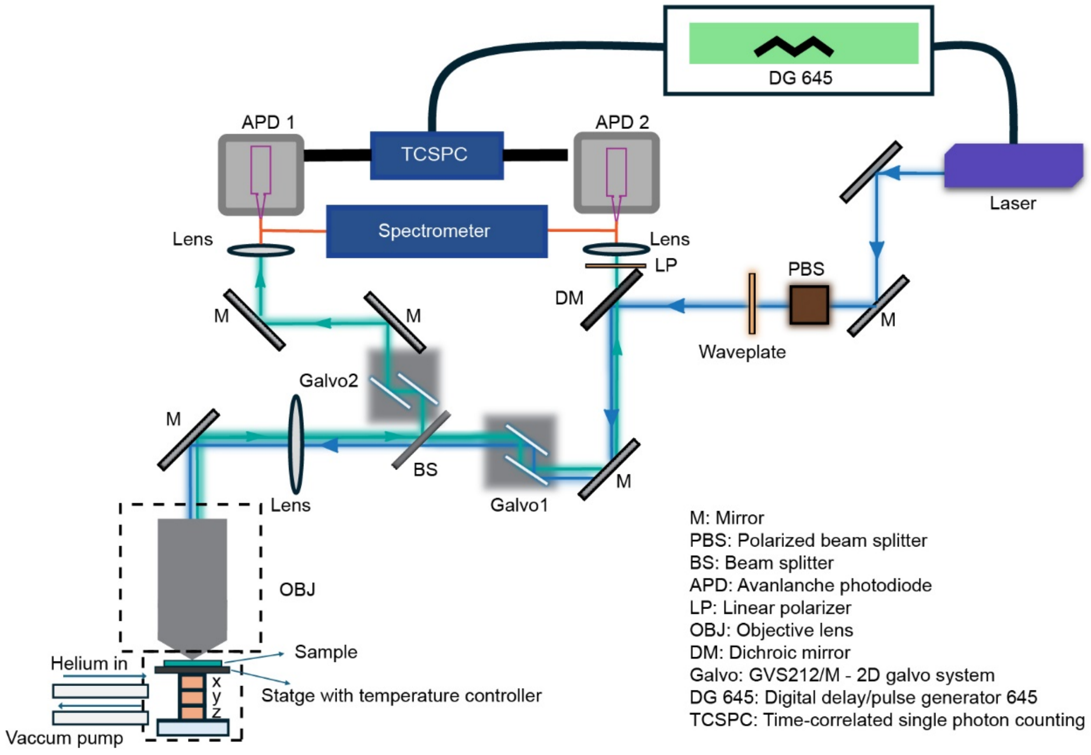

Fig. S11. Schematic diagram of the confocal microscope and measurement setup.

Reference

(1) Yakunin, S.; Protesescu, L.; Krieg, F.; Bodnarchuk, M. I.; Nedelcu, G.; Humer, M.; De Luca, G.; Fiebig, M.; Heiss, W.; Kovalenko, M. V. Low-threshold amplified spontaneous emission and lasing from colloidal nanocrystals of caesium lead halide perovskites. *Nat. Commun* **2015**, *6* (1), 8056. DOI: 10.1038/ncomms9056.

(2) Dong, Y.; Qiao, T.; Kim, D.; Parobek, D.; Rossi, D.; Son, D. H. Precise Control of Quantum Confinement in Cesium Lead Halide Perovskite Quantum Dots via Thermodynamic Equilibrium. *Nano Lett* **2018**, *18* (6), 3716-3722. DOI: 10.1021/acs.nanolett.8b00861.

(3) Krieg, F.; Ochsenbein, S. T.; Yakunin, S.; ten Brinck, S.; Aellen, P.; Süess, A.; Clerc, B.; Guggisberg, D.; Nazarenko, O.; Shynkarenko, Y.; et al. Colloidal $CsPbX_3$ (X = Cl, Br, I) Nanocrystals 2.0: Zwitterionic Capping Ligands for Improved Durability and Stability. *ACS Energy Lett* **2018**, *3* (3), 641-646. DOI: 10.1021/acsenergylett.8b00035.

(4) Ginterseder, M.; Sun, W.; Shcherbakov-Wu, W.; McIsaac, A. R.; Berkinsky, D. B.; Kaplan, A. E. K.; Wang, L.; Krajewska, C.; Šverko, T.; Perkinson, C. F.; et al. Lead Halide Perovskite Nanocrystals with Low Inhomogeneous Broadening and High Coherent Fraction through Dicationic Ligand Engineering. *Nano Lett* **2023**, *4*, 1128–1134. DOI: 10.1021/acs.nanolett.2c03354.

(5) Rainò, G.; Becker, M. A.; Bodnarchuk, M. I.; Mahrt, R. F.; Kovalenko, M. V.; Stöferle, T. Superfluorescence from lead halide perovskite quantum dot superlattices. *Nature* **2018**, *563* (7733), 671-675. DOI: 10.1038/s41586-018-0683-0.

(6) Haug, H. A. K., Stephan W. *Quantum Theory of the Optical and Electronic Properties of Semiconductors*; DOI: 10.1142/7184.

(7) Gantmakher, V.; Levinson, Y. *Carrier scattering in metals and semiconductors*; Elsevier, 2012.

(8) Rhim, J.-W.; Yang, B.-J. Classification of flat bands according to the band-crossing singularity of Bloch wave functions. *Phys. Rev. B* **2019**, *99* (4), 045107. DOI: 10.1103/PhysRevB.99.045107.

(9) Zhu, C.; Boehme, S. C.; Feld, L. G.; Moskalenko, A.; Dirin, D. N.; Mahrt, R. F.; Stöferle, T.; Bodnarchuk, M. I.; Efros, A. L.; Sercel, P. C. Single-photon superradiance in individual caesium lead halide quantum dots. *Nature* **2024**, *626* (7999), 535-541.
